# A Review of the Common Neurodegenerative Disorders: Current Therapeutic Approaches and the Potential Role of Nanotherapeutics

**DOI:** 10.3390/ijms23031851

**Published:** 2022-02-06

**Authors:** Richard N. L. Lamptey, Bivek Chaulagain, Riddhi Trivedi, Avinash Gothwal, Buddhadev Layek, Jagdish Singh

**Affiliations:** Department of Pharmaceutical Sciences, School of Pharmacy, College of Health Professions, North Dakota State University, Fargo, ND 58105, USA; richard.lamptey@ndsu.edu (R.N.L.L.); bivek.chaulagain@ndus.edu (B.C.); riddhi.trivedi@ndsu.edu (R.T.); avinash.gothwal@ndus.edu (A.G.)

**Keywords:** nanoparticle, neurodegenerative disorder, neurogenesis, Alzheimer’s disease, Parkinson’s disease, blood–brain barrier, amyotrophic lateral sclerosis

## Abstract

Neurodegenerative disorders are primarily characterized by neuron loss. The most common neurodegenerative disorders include Alzheimer’s and Parkinson’s disease. Although there are several medicines currently approved for managing neurodegenerative disorders, a large majority of them only help with associated symptoms. This lack of pathogenesis-targeting therapies is primarily due to the restrictive effects of the blood–brain barrier (BBB), which keeps close to 99% of all “foreign substances” out of the brain. Since their discovery, nanoparticles have been successfully used for targeted delivery into many organs, including the brain. This review briefly describes the pathophysiology of Alzheimer’s, Parkinson’s disease, and amyotrophic lateral sclerosis, and their current management approaches. We then highlight the major challenges of brain-drug delivery, followed by the role of nanotherapeutics for the diagnosis and treatment of various neurological disorders.

## 1. Introduction

Neurodegeneration has been identified as the pivotal pathophysiological change in most brain-related disorders [1]. Regardless of the incessant efforts by modern science to create a medical or surgical solution, the outcome has not been favorable. Neurodegenerative disorders (NDs) such as Alzheimer’s and dementia continue to be a clinical concern in most older people [2,3]. The highly effective blood–brain barrier (BBB) continues to be a real barrier towards the successful management of NDs. Despite the several successes that have been demonstrated with surgeries and highly evasive techniques, their clinical acceptance is limited due to varying concerns about their long-term benefit, owing to the potential damage to the brain barrier. As a suitable alternative for halting or reversing neurodegeneration, nanotherapeutics with the potential to cross the BBB (without damage to the barrier) have been proposed and demonstrated in many cases [4,5]. Nanotherapeutic use is gaining traction due to the several benefits compared to conventional dosage forms [6]. Despite this great progress, there is a need to refine nanotherapeutics to ensure optimum outcomes. In this review, we initially describe the pathophysiology of major NDs and their current management strategies. We also discuss the role of BBB and other challenges for brain-targeted drug delivery. Further, we look at the potential role of nanotherapeutics in the fight against neurodegeneration. Finally, we discuss breakthroughs and current findings in nanotherapeutics to manage NDs and provide perspectives for future applications.

## 2. Neurodegenerative Disorders (NDs)

**Figure 1:**
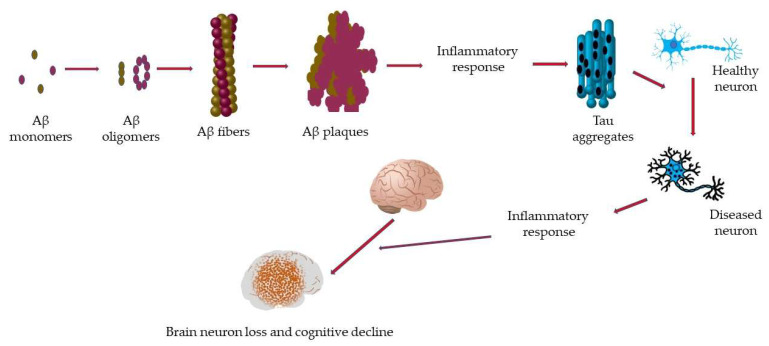
Path to cognitive decline in neurodegeneration. Amyloid-beta (Aβ) monomers clump together to form oligomers of variant structures. Subsequently, the oligomers aggregate to form Aβ fibers, which misarrange to form Aβ plaques. Plaque formation induces an inflammatory response which includes the formation of tau aggregates leading to the conversion of healthy neurons to diseased neurons. The presence of more diseased neurons triggers another inflammatory response leading to more neuron loss and a subsequent loss in brain function as well as cognitive decline.

Neurons are central to the proper functioning of the human brain since they play a critical role in communication [7,8]. Most neurons originate in the brain; however, neurons are present everywhere in the body [9,10]. During childhood, neural stem cells produce the majority of neurons, the number of which is significantly reduced in adulthood [11]. Although neurons are not immortal, the progressive loss of neurons, neuron structure, and/or their functions, known as neurodegeneration, is central to the pathophysiology of several brain disorders [12] and is also a major health concern. Neurodegeneration is associated with dysfunction of the synapse, neural network, and the deposition of physiochemically altered variants of proteins in the brain (Figure 1) [13,14,15,16]. Diseases with neurodegeneration as their hallmark feature are collectively termed NDs [17,18]. The most common NDs include Alzheimer’s disease, Parkinson’s disease, prion disease, Amyotrophic lateral sclerosis, motor neuron disease, Huntington’s disease, spinal muscular atrophy, and spinocerebellar ataxia [17,19,20,21].

Neurodegenerative disorders affect millions of people worldwide. Although age is the single most contributing risk factor to the development of all NDs, recent findings reveal that a combination of an individual’s genetic makeup and environmental factors can equally contribute to increasing the risk for NDs. Further, despite the expression of specific genes (within an individual) accountable for NDs [22], the time and extent of neurodegeneration largely depend on their immediate environment [23,24]. More recent studies reveal that multiple pathologies may underline a single neurodegenerative disorder [25,26,27,28]. Thus, NDs can be very serious or even, in certain instances, life threatening; however, it solely depends on the type and stage of the disease.

Since the brain controls several aspects of the body’s function, neurodegenerative diseases consequently affect multiple facets of human functioning and limit the ability to perform both basic (e.g., speech, movement, stability, and balance) and complicated tasks (e.g., bladder and bowel functions, and cognitive abilities). Most NDs progress without remission, whilst in some cases, treatments target the improvement of symptoms, relief of pain if present and/or the restoration of balance and mobility. In the following sections we will briefly discuss some common NDs.

### 2.1. Alzheimer’s Disease (AD)

More recent studies of Alzheimer’s disease pathophysiology have shown that the accumulation of amyloid-beta (Aβ) and tau proteins are central to AD progression [29,30]. The formation of Aβ-containing plaques within the brain, linked with neurofibrillary tangles (NFTs) composed of hyperphosphorylated tau, has been identified as the classical feature of AD [31,32,33]. Plaque formation disrupts hippocampal circuitry leading to poor short-term memory consolidation into long-term traces [34]. In AD, there is extensive neuronal loss, faulty synaptic connections, and damage to the essential neurotransmitter systems necessary for brain functions, including memory. Thus, the most common clinical symptom in early-stage AD is selective memory impairment. In addition, hippocampus and medial temporal-lobe-dependent functions, such as declarative episodic memory, are also often affected. Finally, executive function impairment, judgment, and problem-solving are additional clinical manifestations and usually appear early [35]. 

### 2.2. Parkinson’s Disease (PD)

Parkinson’s disease is a progressive neurological disorder that leads to tremors, muscle stiffness, an unsteady walk, and balance and coordination difficulties. Both genetic and non-genetic stimuli cause PD. Age is considered the primary risk factor for PD [36,37]. In addition, several other factors, such as excessive caffeine intake, smoking, and exposure to environmental toxins, are known to modulate the risk of development of PD [38], although the exact mechanism remains unclear [39,40,41]. The pathophysiology of PD primarily includes frontal cortex atrophy and ventricular enlargement. However, the most distinctive morphological alteration observed in the PD brain is the loss of pigmentation in the locus coeruleus and substantia nigra pars compacta (SNpc), which stems from the death of dopaminergic (DA) neuromelanin-containing neurons [42]. In PD, this significant cell loss results in dysfunction of the nigrostriatal pathway, culminating in decreased dopamine concentration within the striatum, and consequently, the cardinal motor symptoms [42]. Cell loss in different regions, including the nucleus basalis of Meynert, the raphe nuclei, the locus coeruleus, the pedunculopontine nucleus, the dorsal motor nucleus of the vagus nerve, the hypothalamus, and the olfactory bulb, account for the non-motor symptoms of PD [43].

Several mechanisms have been identified to play key roles in PD disease progression, and these include α-synuclein misfolding and aggregation, mitochondrial dysfunction, dysfunctional protein clearance systems, the ubiquitin–proteasome system, and autophagy–lysosome system, and neuroinflammation [37,42,44].

Microscopically, the presence of Lewy bodies (abnormal cytoplasmic deposits which are immunoreactive for the protein α-synuclein) within neuronal cell bodies, accompanied by dystrophic neurites (Lewy neurites), characterize PD [36,45]. Lewy bodies may be phosphorylated and spread to other regions of the CNS. Similar to AD, protein misfolding also occurs in PD [45], and the protein that is commonly misfolded is the tau protein. The abnormal hyperphosphorylation of tau protein leads to NFT formation. In a subgroup of PD patients, there are widespread plaques of NFTs and amyloid-beta plaques [46].

### 2.3. Amyotrophic Lateral Sclerosis (ALS)

ALS, more commonly referred to as motor neuron disease or Lou Gehrig’s disease, is a progressive disease of the nerve cells and spinal cord, resulting in muscle weakness and paralysis [47,48]. In ALS, motor neurons gradually deteriorate before they die [49]. When motor neurons are damaged or dead, signals that should be sent to the brain are no longer delivered. Although over 30 different genes have been associated with ALS, mutations in four main genes (C9orf72, TARDBP, SOD1, and FUS) account for greater than 70% of ALS cases [49]. These four genes encode for proteins involved in major motor function aspects such as DNA repair, homeostasis, mitochondrial function, and glial cell function. A combination of these impaired functions is believed to contribute to the degeneration of motor neurons observed in ALS. Accumulation of intraneuronal protein aggregates is the pathological hallmark of ALS. The most abundant protein observed in most ALS patients is the TAR DNA binding protein; however, other proteins such as superoxide dismutase-1 and neurofilament can also form aggregates [50,51]. Nonetheless, it is unclear whether protein aggregates or protein complexes precede neuron damage or vice versa.

## 3. Current Therapeutic Approaches to Treat ND

Management of neurodegenerative disorders is often disease-specific. Several approaches to management are currently accepted, which either target the disease pathogenesis or attempt to improve the symptoms experienced. In this review, we consider the therapeutic approaches currently in practice to treat major NDs (Table 1).

### 3.1. Therapeutic Approaches for AD

Therapeutic approaches for managing AD focus mainly on targeting different pathways for disease progression. Currently, there are three classes of drugs approved by US-FDA for the management of AD, each of which is described below.

#### 3.1.1. Antibody Targeting Amyloid-Beta (Aβ) Plaques

Aducanumab (Aduhelm) is the first disease-modifying drug approved for AD patients, and was approved in June 2021 [52]. It is administered as an intravenous (IV) infusion over approximately one hour every four weeks. Aducanumab is an IgG1 monoclonal antibody specific to extracellular Aβ plaques in the brain, which binds and helps in clearing the plaques [52,53]. Although conditionally approved, clinical data on aducanumab show a reduction in the Aβ plaques’ load, but with no relationship to improved cognitive function in patients. More clinical data will still be collected to provide conclusive evidence of whether the drug helps in cognitive functions. However, the approval of aducanumab has also created a wave of excitement in AD patients and advocacy groups. Besides being the first therapy to target altering the pathology of the disease, they believe it will create avenues for similar therapies in the near future. 

Multiple clinical trials have been performed using different bioactive molecules (i.e., secretase inhibitors and therapeutic antibodies), but most of them have terminated so far. Some Aβ targeting antibodies—AAB-003, MEDI1814, RO7126209, and SAR228810—have completed clinical trial phase I. While aducanumab has completed clinical trial phase III, it is also specific towards Aβ aggregation. Similarly, tau or TREM 2 specific antibodies, i.e., BIIB076, bepranemab, JNJ-63733657, have completed clinical trial phase I, while gosuranemab is in clinical trial phase 2 [54]. Thus, additional antibody-based targeting medicine may obtain FDA approval for AD treatment shortly.

#### 3.1.2. Cholinesterase Inhibitors

Currently, cholinesterase inhibitors are the first-line medications administered for AD. Donepezil, rivastigmine, and galantamine are the three main cholinesterase inhibitors used clinically. In AD, there is an associated loss of cholinergic neurons and recession in the quantity of acetylcholine in the cortical regions of the brain. Multiple studies have revealed that the increased supply of acetylcholine in demented patients helped diminish the cognitive decline. Cholinesterase inhibitors limit acetylcholine degradation, and the patient benefits from increased cholinergic activity [55]. 

Tacrine was the first choline esterase inhibitor approved back in 1993 by the FDA, but was discontinued later because of its associated hepatotoxicity. Donepezil is used for mild-to-moderate AD and is administered as oral tablets of 5 or 10 mg/day. More recently, higher doses of donepezil (23 mg/day), alone or combined with memantine, were approved for moderate-to-severe patients. Another acetylcholinesterase inhibitor used for mild-to-moderate AD is rivastigmine. Unlike other cholinesterase inhibitors, rivastigmine is available as a transdermal patch and inhibits both acetylcholinesterase and butyrylcholinesterase enzymes. Galantamine, the next class of cholinesterase inhibitors, was approved for mild-to-moderate AD at a dose range of 16–24 mg/day. Besides its inhibitory effect on cholinesterase activity, it also produces allosteric modulation of nicotinic cholinergic receptors [56].

Although numerous drugs have been developed for AD, cholinesterase inhibitors remain the only option available to patients. The recently approved drug aducanumab is contentious in its efficacy and exorbitant in pricing. However, cholinesterase inhibitors show limited efficacy. They achieve a modest improvement in patients’ cognitive ability, and they are labeled as symptomatic treatment options rather than altering the pathology [57]. Moreover, questions exist on whether current medications can effectively cross the BBB at significant doses to elicit the desired pharmacological effects.

#### 3.1.3. Glutamate Regulators

Glutamate is the major excitatory neurotransmitter in the brain. Through excessive activation in postsynaptic neurons such as NMDA receptors, glutamates confer neuronal damage, leading to neurodegeneration. However, complete inhibition of NMDA receptors has resulted in severe side effects. Consequently, memantine, an uncompetitive NMDA receptor antagonist, was developed, which provides pathological benefits with NMDA receptor activation and also protects patients from inhibitory effects due to overactivation [58]. 

Memantine was approved in 2003 for moderate-to-severe AD patients at a dose of 5–20 mg/day. Monotherapy of memantine benefitted AD patients with improved cognition over a placebo. In combination with acetylcholinesterase inhibitors, clinical trials showed improved efficacy for one year over monotherapy [59,60]. However, this class of drugs also fails to address the pathology of AD, and is mainly used to alleviate symptoms. 

### 3.2. Therapeutic Approaches for PD 

Although PD is the second most common ND, there is a lack of effective therapy that especially alters the pathophysiology of the disease. Instead, some options address the motor-related symptoms and non-motor-related symptoms separately for symptomatic relief in patients. 

The principal approach in managing PD is to replenish the decreased dopamine levels in the substantia nigra region of the brain. Numerous approaches have been put in place that aim to replenish dopamine levels. The most common therapy is a combination of levodopa and carbidopa. Levodopa is an immediate precursor of dopamine that helps restore motor functions resulting from the loss of dopamine. Carbidopa is combined with levodopa to inhibit the peripheral breakdown of levodopa before it reaches the brain. Additionally, entacapone and tolcapone are also used to prevent methylation of levodopa through catechol-O-methyl transferase (COMT), thereby preventing levodopa loss through methylation.

Dopaminergic agonists such as apomorphine hydrochloride, pergolide, pramipexole dihydrochloride, ropinirole hydrochloride, and rotigotine, which produce an identical effect to dopamine, are also available for the treatment of PD. Monoamine oxidase inhibitors are the next class of drugs available. They inhibit the oxidative deamination of dopamine in the brain and prevent dopamine loss. Selegiline and rasagiline are the two examples of monoamine oxidase inhibitors [39].

PD is associated with major non-motor symptoms (MNMS) like all NDs, including depression, psychosis, sleep disorders, constipation, dementia, and olfactory deficit, and they are treated symptomatically [61]. Rivastigmine, donepezil, and galantamine are prescribed to alleviate dementia-related symptoms. Clozapine, quetiapine, and pimavanserin are approved medications for treating psychosis-related symptoms. The use of melatonin or clonazepam is suggested for sleep-related symptoms [62].

### 3.3. Therapeutic Approaches for ALS

ALS is a motor neuron disease that manifests the symptoms of frontotemporal dementia, behavioral changes, and cognitive decline with the progression of the disease. ALS patients succumb to respiratory failure and death within three to five years of the appearance of symptoms [63]. There are two approved medications for ALS patients, i.e., riluzole and edaravone. Riluzole, a glutamate-receptor antagonist, was approved back in 1995 as an oral tablet with a dose of 100 mg/day. Clinical trials have shown that riluzole use prolongs the life of ALS patients by 3 to 4 months compared to a placebo group. Edaravone, a free-radical scavenger, was recently approved in 2017 as an intravenous infusion with a dose of 60 mg/day, and it helps to delay the progression of the disease [64]. Besides these medications, patients are treated symptomatically for the improvement in their quality of life. There are no concrete disease-modifying therapies available as of now. 

## 4. Challenges of Brain-Drug Delivery

Current therapy for the management of NDs has aided in controlling the progression of the disease rather than eliminate the root causes. The problem of neurodegeneration lies behind the BBB, and that is where most of these formulations fail. The inability to transport sufficient doses to the brain limits the successful intervention of NDs. The advanced nature of the BBB, coupled with the poor permeative potency of most, if not all drugs, accounts for the lack of suitable treatment options for NDs.

### 4.1. The Blood–Brain Barrier (BBB)

The BBB has been described as a diffusion barrier that prevents substances in the blood from entering the brain, allowing the maintenance of homeostasis and the brain’s normal functioning [65]. Different cells in the brain (brain microvascular endothelial cells, tight junctions, neurons, astrocytes, and basal membranes) fuse to build a physically tight brain capillary in the BBB [66]. The absence of fenestrations within the brain capillary endothelial cells limits the diffusion of small molecules and proteins [67,68]. The endothelial cells are further linked to a continuous barrier through inter-endothelial junctions, restricting the transport of water-soluble substances [69,70]. Furthermore, the endothelial cells are surrounded by the basal lamina, astrocytes, and pericytes, limiting access to drug molecules from the blood to the brain [71]. The strength of this barrier is complimented by efflux transporters located in the brain capillary, and these transporters return substances that enter the brain back into the bloodstream [72]. In addition, the permeability function of the BBB is further regulated by intere-ndothelial junctions, which are protein complexes including tight junctions, gap junctions, and adherens junctions [65,73]. Molecules that cross the BBB either go through the paracellular or transcellular pathway [73]. The physicochemical properties of compounds that allow their transport across the BBB include the size, molecular weight, surface activity, lipid solubility, and charge [74,75]. Some small molecules (such as ethanol, carbon dioxide, and barbiturates) freely cross the BBB through passive diffusion [76,77]. Receptor-mediated transport mechanisms including the insulin transporter, transferrin receptor, and glucose transporter-1 (GLUT-1) [78] also aid the transport of hydrophilic molecules such as peptides and proteins [76]. Further, some pathologic states are known to disrupt the tightness of the BBB, allowing the leakage of substances into the brain [79,80,81,82]. Finally, the use of specialized drug carriers such as nanoparticles can enhance the transport of cargos across the BBB. A few of these instances are discussed below.

### 4.2. Pharmacokinetic Principles and Their Effects on Brain-Drug Delivery

The efficacy of systematically administered drugs is mostly determined by their pharmacokinetic characteristics [83]. From the point of administration to the target site (in this case, the brain) is a harrowing journey that, in most cases, does not favor the therapeutic molecules. The first point of attention is the presence of various plasma proteins that are embedded within. Some drugs are highly bound to these proteins, thereby limiting the amount of the drug available in circulation, ultimately reducing the free drug available for transportation to the brain [84]. Additionally, some drugs are uneliminated by the major clearance organs at a significant rate, leaving only a few within the bloodstream. In addition, the interaction between drug and target cells limits the extent of drug absorption. More specifically, drug molecules can affect cells that lead to the blocking of channels, a change in membrane potential, or even an alteration in cell conformation. This transient effect can limit the behavior of the cell towards the administered drug molecule and its absorption [85]. In general, small lipophilic drug molecules are suitable for brain delivery [86].

## 5. Nanoparticles and Their Use in NDs

Limitations caused by the BBB and the disadvantages of the current therapies, as mentioned above, have led to the unmet need for new therapeutic approaches for the treatment of NDs [87]. Out of the approaches employed, nanotechnology has emerged as a safe and promising platform for targeted drug/gene delivery to the CNS [88,89]. This technology employs materials in nanoscale, usually ranging from 1–1000 nm, and can interact with biological systems at the molecular level [90]. A variety of materials such as natural polymers (proteins and polysaccharides), synthetic polymers (PLGA and PCL), and inorganic materials (gold, silver, and cerium) have been employed to formulate nanoparticles. Nanocarriers have proven to be highly suitable drug/gene carriers to the brain [91]. The characteristics of nanocarriers that make them a promising platform for managing and treating NDs include high drug loading capacity, low systemic toxicity, improved drug permeabilization, and good physical and chemical stability [88]. 

**Figure 2:**
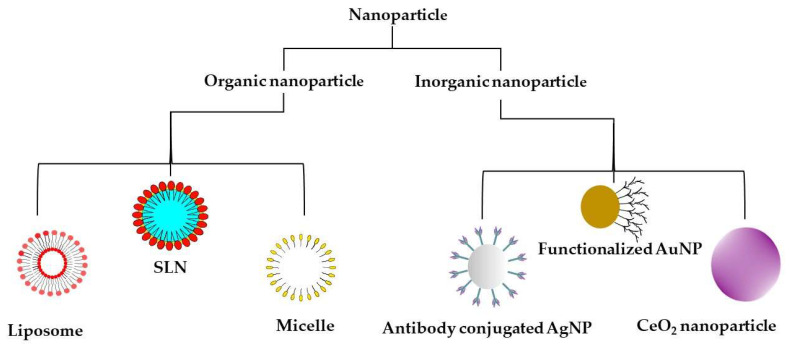
Types of nanoparticles most commonly used for the management of neurodegenerative disorders. SLN—solid lipid nanoparticle; AuNP—gold nanoparticle, AgNP—silver nanoparticle, CeO_2_ NP—cerium oxide nanoparticle.

Nanoparticles with varying sizes, properties, and functions have been developed for brain-drug delivery; their forms are provided in Figure 2. However, their penetration through the BBB depends on the size, surface chemistry, type, and polarity of the nanocarriers [92]. Additionally, the surface coating with polysorbate can help evade transmembrane efflux systems such as P-glycoprotein pumps [92]. Liposomal and polymeric nanoparticles have been the most exploited for targeted brain delivery due to the ease of surface modification with ligands and cell-penetrating peptides (CPPs) [78,93,94,95,96]. 

Here, we discuss the nanoparticles most commonly studied for the management of NDs. 

### 5.1. Inorganic Nanoparticles 

Metal nanoparticles have gained much interest due to their ability to easily cross the BBB and accumulate in the brain [97,98]. Their various properties, such as size, surface modifications, and stability, can be easily modulated for efficient brain targeting [98]. For instance, metal nanoparticles are often functionalized with various brain-targeted ligands, such as antibodies, proteins, and small molecules (e.g., mannose) for enhanced drug delivery to the CNS. These nanoparticles are also widely known for their theragnostic and imaging applications [99,100]. Among various metallic nanoparticles, gold, silver, and cerium nanoparticles have been the most exploited for CNS delivery [97] and will be discussed here.

Gold nanoparticles (AuNPs) have been extensively used in CNS imaging and targeting [101]. Their core has plasmonic properties (i.e., the ability to interact with electromagnetic radiation due to the presence of free electrons), making them ideal for imaging applications using micro-CT scanning or X-rays. The AuNPs are superior in absorbing and reducing the X-rays better than the conventional contrast agents, which allows for higher contrast and precise visualization of the nanoparticles [100]. In a recent study, rhodamine B isothiocyanate (RITC) and poly-L-lysine (PLL) were complexed with 40 nm AuNPs. These modifications increased nanoparticle uptake in human mesenchymal stem cells (hMSC). This gold-labeled hMSC was directly injected into rat brains and could be visualized 30 min post-injection using the micro-CT [102]. In combination with cell tracking and visualization, AuNPs have shown great potential in targeting and degrading β-amyloid aggregates under in vitro conditions [103]. Apolipoprotein E3 (ApoE3) conjugated with the core of the AuNPs, promoted their interaction with the amyloid aggregates and increased penetration in the brain. Curcumin was used as a probe to track these AuNPs. Upon binding of amyloid aggregates and ApoE3-AuNPs, the surface plasmon resonance (SPR) of the AuNPs was used to dissociate the amyloid aggregates by 60% [104]. In another study, AuNPs were surface-modified with brain-targeted exosomes for more effective and enhanced brain delivery [105]. Sub-cellular size, unique size-dependent physicochemical and optical properties, adaptability, and biocompatibility of AuNPs make them suitable carriers for brain-targeted delivery of small molecules and biomacromolecules [106]. In a different report, gold nanoparticles differentiated mouse embryonic stem cells into dopaminergic neurons [107]. 

Silver nanoparticles (AgNPs) have also been explored for brain-targeted drug delivery. After intraperitoneal injection, the AgNPs reached and accumulated in the hippocampus, which is known to be an essential region for NDs [108]. It was noted that a 5µg/mL dose of these nanoparticles could induce an inflammatory and neurodegenerative gene expression response in mice’s neural cells [98,109]. AgNPs have been used to deliver a myriad of drugs to the brain, ranging from alisertib for glioblastoma [106] to anti-amoebic drugs to treat brain-eating amoebae [110]. Another study highlighted the anti-inflammatory and antioxidant properties of citrate-capped AgNPs on microglia, the brain’s immune cells. These AgNPs were absorbed specifically by microglia, which in turn led to the expression of enzymes that reduced reactive oxygen species and had anti-inflammatory properties [111]. However, a drawback evidenced with AgNPs is their mechanism of entry into the brain, which involves disruption of the BBB by weakening the tight junctions. They also seem to induce neuronal degeneration and necrosis by accumulating inert silver in the brain over a long period [112,113,114]. 

Cerium oxide nanoparticles are most known for their role in reducing oxygen species (ROS), linked to neuronal death and NDs. The transition of oxidation states between Ce^+3^ and Ce^+4^ is the reason behind the excellent antioxidant property these nanoparticles display [115]. These nanoparticles have proven to retard the apoptotic effect of AD in neuronal cells by altering the brain-derived neurotrophic factor signal transduction pathway, and showed the potential to decline Aβ-aggregation when combined with PEG coatings or metal chelators [116]. Besides this, cerium oxide nanoparticles have effectively scavenged peroxynitrite ROS in ischemic stroke models and restored limb motor function in multiple-sclerosis- and ALS-mouse models [117]. Recently, several nanoparticle-based formulations have been documented with neurogenesis potential. Zavvari et al., 2020 explored the neurogenesis efficacy of cerium dioxide (CeO_2_) nanoparticles. They claimed that single-dose administration of CeO_2_ nanoparticles was enough to initiate neurogenesis in the hippocampal region. This is due to the anti-inflammatory and neuro-regenerative potency of cerium oxide [118].

Several documented reports confirm behavioral or functional improvements in vivo when treated with nanoparticles. For example, Wu et al., 2020 explored that iron-oxide-nanoparticle-tagged MNCs were able to migrate from the administered site to the choroid plexus and trigger a functional recovery in the ischemic-stroke brain. The author suggested that MNCs could be more beneficial if administered directly into the lateral ventricles instead of intravenously [119].

### 5.2. Organic Nanoparticles 

Naturally occurring molecules, such as lipids and other organic molecules, can be exploited as tools for delivering nanomedicine due to their superior biocompatibility compared to inorganic materials. Moreover, a lipid nanocarrier is more effective in protecting the therapeutic moiety from degradation, reducing toxicity and increasing biocompatibility, than the free-drug administration [116]. Among the different lipid carriers, liposomes have been the most extensively explored for brain-targeted delivery. Liposomes dual functionalized with mApoE and phosphatidic acid were developed to enhance delivery across the BBB and target Aβ aggregates with high affinity [120]. This liposomal formulation could disaggregate Aβ fibrils in vitro. The negatively charged phosphatidic acid interacts with the positively charged amino acid residues on the Aβ, while the mApoE interacts with the negatively charged regions of the same. 

In a recent study, our lab has developed surface-modified liposomes for brain-targeted delivery of ApoE2-encoding plasmid DNA [121]. The targeting ligand used was mannose along with a CPP (penetratin and rabies virus glycoprotein peptide, RVG) to enhance brain-targeting and cellular internalization, respectively. Similarly, liposomes modified with RVG and transferrin displayed superior uptake in brain endothelial cells, astrocytes, and neurons as compared to plain liposomes [94]. In a separate study by Rodriguez et al. [93], surface functionalization of liposomes with transferrin and a CPP was sufficient to improve the brain permeability of liposomes in mice after a single intravenous administration. In all of these studies, drug accumulation in the brain was attributed the surface functionalization.

**Figure 3:**
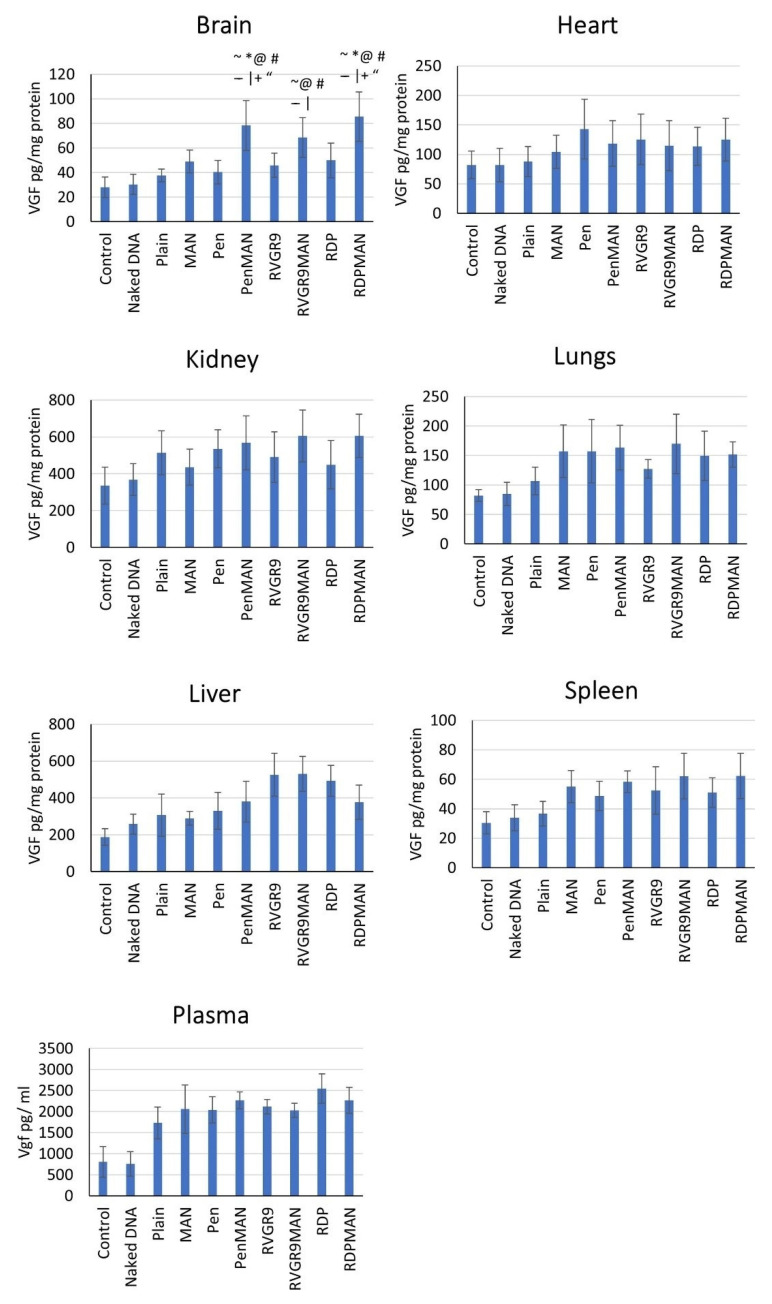
In vivo vgf transfection in brain and other major organs in mice. Data shown as mean ± SD of 6 animals per group. ~, |, @, #, *, –, +, and “ show statistically significant difference (*p* < 0.05) from control, naked DNA, plain, Pen, MAN, CGN, RVG9R and RDP liposomes, respectively. Source: Reprinted from Arora, S.; Singh, J. In vitro and in vivo optimization of liposomal nanoparticles based brain targeted vgf gene therapy. International Journal of Pharmaceutics 2021, 608, 121095 [122]. With permission from Elsevier.

Similarly, optimized brain targeting liposomes, functionalized with mannose and either RVG, penetratin, rabies-derived peptide (RDP), or CGNHPHLAKYNGT (CGN) peptide sufficiently delivered VGF (VGF nerve growth factor inducible) across in vitro BBB models and in vivo mouse models. In this study the authors observed a 1.5–2.0-fold (*p* < 0.05) higher transfection in functionalized-liposome-treated mice compared to an untreated control-mouse group (Figure 3). Further, the formulated liposome nanoparticles were biocompatible both in vivo and in vitro [122].

Antioxidants can protect the neurons from amyloid-β-plaque-mediated oxidative damage. Curcumin has displayed promising antioxidant potential against various NDs [123]. It binds to the Aβ deposits, disrupts aggregation, and disaggregates pre-formed fibril, both in vitro and in vivo [124,125]. Besides liposomes, solid lipid nanoparticles (SLN) have also been used for brain-targeted delivery of therapeutics to manage various NDs. Rosmarinic-acid-loaded SLNs were administered intranasally to ameliorate the behavioral dysfunctions and oxidative stress associated with Huntington’s disease [126].

Nanomicelle, particularly polymeric nanomicelle, has emerged as a potential vehicle to deliver diverse therapeutic agents [127,128]. Depending on their hydrophilic and hydrophobic characteristics, polymers that self-assemble to form micelles can do so at reasonably lower concentrations while maintaining a small internal diameter, sufficient to carry cargo [129]. More recently, some studies have demonstrated the ability of functionalized chitosan nanomicelles to transfect the brain cells at effective concentrations [130]. Chitosan nanomicelles present the advantages of being biodegradable, nontoxic at the concentration of use, and flexible towards surface modification [131]. These advantages make chitosan nanomicelles an excellent carrier for delivering drugs, proteins, genes, and even antibodies to the brain. Recently, Xue et al. [132], conjugated chitosan nanoparticles significantly inhibited by α-syn aggregation in vitro, as well as discovering significant neuroprotective effects in Parkinson disease models. Chitosan can equally be used in conjugation with other polymers to enhance delivery across the BBB. In a separate study by Jaruszewski et al. [133], Chitosan-coated PLGA nanoparticles had a better BBB uptake compared to naked PLGA nanoparticles.

While different polymeric formulations have been employed in the production of nanoparticles, poly D, L-(lactic-co-glycolic) acid (PLGA) has been extensively used for brain-targeted and controlled drug delivery [134]. This biodegradable, biocompatible polymer, with adjustable degradation rates, a high drug loading capacity, and the ability to cross through the BBB to target the brain, makes it an ideal carrier system for treating NDs. In one study, TET1 peptide-coated PLGA nanoparticles were used to encapsulate and deliver a hydrophilic drug, nattokinase, to the brain. The TET1 peptide demonstrated a high affinity for neurons and promoted retrograde transport. This formulation successfully improved the stability of the nattokinase protein and downregulated amyloid aggregation, proving to be a vital option for treating AD [135]. A separate study by Carradori et al. synthesized Anti Aβ1-42 conjugated poly (alkyl cyanoacrylate) nanoparticles directed toward Aβ1-42. When transgenic AD mice were treated with these nanoparticles, there was a significant decrease in the brain and plasma level of Aβ soluble peptide and its oligomer, resulting in corrected memory defect [136]. 

In a separate study by Safari et al., phosphatidylserine nanoliposomes also improved the memory of AD-induced rats when loaded with metformin. In this study, IL1-β, TNF-α, and TGF- β levels were found to be reduced in the hippocampal region. Neurogenesis was observed along with significantly reduced necrosis and neuroinflammation [137]. 

As we have discussed, nanoparticles are capable of initiating neurogenesis in in vivo systems, but nanoparticles have also been explored to aid stem cells’ differentiation into neurons. For example, polycaprolactone-lignin nanoparticles triggered neurogenesis and neurite outgrowth in PC12 and hADSCs cells. The developed nano-scaffold was biocompatible and safe. The author claimed that the incorporation of 15% lignin nanoparticles improved the expected outcomes: neuro-construction and regeneration [138]. Similarly, NGF-loaded chitosan nanoparticles differentiated canine mesenchymal stem cells into neuronal cells [139].

Similarly, RA-NPs improved neuronal cell differentiation, survival, and viability in neural stem cells after the ischemic effect [113].

## 6. Nanomedicines under Clinical Trial

There is an utmost need to develop novel treatment strategies against neurodegenerative disorders, that pause neurodegeneration rather than provide symptomatic relief. Several studies on nanoparticles, show promise of an effective drug delivery approach, which can be a ray of hope against neurodegenerative disorders.

A recent search in ongoing clinical trials revealed less than 10 nanoparticle-based formulations under different phases of clinical trials against NDs (Table 2). Only one clinical trial of lipid nanoparticle-based formulation for transthyretin-mediated amyloidosis has been completed and approved for sale in public. While a (CRISPR)/Cas9 gene-based study is in the clinical trial phase I, lipid nanoparticles are being used as a drug delivery platform for this study. An exciting approach of nanoparticle-mediated delivery of APH-1105 against mild-to-moderate AD is enlisted; this clinical trial will be started in 2023. However, a gold-nanoparticle-mediated CNM-Au8 delivery approach is in the clinical trial phase 2. On the other hand, multiple studies of CNM-Au8-gold nanocrystals-based studies are in phase 1 and phase 2 of clinical trials against ALS.

## 7. Challenges, Future Prospects and Conclusions

Neuronal death is the primary characteristic of NDs, i.e., AD and Parkinson’s. Therefore, neurogenesis is the most envisioned treatment strategy for these disorders. However, drug delivery to the brain is still a challenge due to multiple crucial factors, including the BBB, lipophilicity, the molecular weight of the drug, etc. These factors limit the therapeutic potency of drugs and make NDs more challenging to treat. Thus, nanoparticle-mediated targeted drug delivery to the brain has been explored in recent years for neurogenesis, and it provides a promising platform for improving treatment strategies. Despite these potential advantages, nanocarrier-mediated drug delivery has some challenging aspects, including safety, production, and regulations. 

The toxicity of nanoparticles primarily depends on size, surface charge, ionic dissolutions, and shape. These features should be considered for developing nanoparticle-based drug-delivery systems as per the official nanotoxicity guidelines [140]. Additionally, the approval of these nanocarriers should be critically examined, including the effects on health and the environment [141]. A great pool of literature suggests several amendments to minimize the toxicity associated with the size and charge, such as surface modification with biodegradable- or bio-molecules of intrinsic origin [142,143]. As far as the production of nanocarriers is concerned, they should maintain batch-to-batch uniformity in terms of their size and content. There are multiple methods documented for nanoparticle production, including high-pressure homogenization, microemulsion, extrusion etc. 

Furthermore, the pharmacokinetic properties of the nanotherapeutics greatly impact its efficacy and toxicity. Therefore, it is crucial to investigate the pharmacokinetic parameters of nanotherapeutics in a relevant animal model. In this regard, Pharmacokinetic and more advanced physiologically based pharmacokinetic models can be utilized as a potential tool to predict the in vivo nature of nanotherapeutics [144]. Additionally, regulatory requirements for the clinical acceptance of nanotherapeutics should be considered critically [145]. 

Nanomedicine is a ray of hope for NDs, and it can be an effective tool to overrule the barriers of current and traditional treatment approaches [146]. We highlighted nanoparticle-based reports against various NDs, which may open the prospect of nanomedicine. Understandably, the development of curative treatment is not an immediate process, but preliminary research in the field may lead to a steppingstone that can help eradicate NDs. However, to prove the efficacy against NDs, the generation of more in vitro and in vivo data is needed. Furthermore, thorough in vitro and in vivo investigations and their correlation establishment are required to assess the efficacy of nanoparticles. This would help the research fraternity to extend or identify the effective nanoparticles for diagnostic or therapeutic applications.

## Figures and Tables

**Table 1:** Current therapeutic approaches for the management of neurodegenerative disorders.

Neurological Disorder	Drug Class	Mechanism	Drugs
Alzheimer disease	Amyloid-directed antibody	Acts by targeting and removing amyloid-beta plaques	Aducanumab
	Cholinesterase Inhibitors	Prevent the knockdown of acetylcholine	Donepezil, rivastigmine, galantamine
	Glutamate regulators	Antagonize N-methyl-D-aspartate (NMDA) receptor to improve signal-to-noise ratio of glutamatergic transmission	Memantine
Parkinson disease	Dopamine supplements	Replenish the decreased dopamine levels	Levodopa
	Decarboxylase inhibitors	Prevent peripheral breakdown of levodopa	Carbidopa
	Dopamine agonist	Produces dopamine-like effects	Apomorphine hydrochloride, pergolide, pramipexole dihydrochloride, ropinirole hydrochloride, rotigotine
Amyotrophic Lateral Sclerosis	Glutamate-receptor antagonist	Inhibits glutamate receptors	Riluzole
	Free-radical scavanger	Scavanges free radicals	Edaravone

**Table 2:** Nanocarrier-mediated formulation under clinical trials against different neurodegenerative disorders (ClinicalTrials.gov accessed on 31 January 2022).

Product (Active Molecules/Class)	Nanocarrier (Composition)	Indications	Clinical Phase, NCT Number
ALN-TTR02 (Patisiran)	Lipid nanoparticle (DLin-MC3-DMA; PEG2000-C-DMG; DSPC; and cholesterol)	Transthyretin mediated amyloidosis	Approved for marketing, NCT02939820
APH-1105 (an α-secretase modulator)	Nanoparticle	Mild-to-moderate AD, dementia	Phase 2, NCT03806478
Short palindromic repeats (CRISPR)/Cas9 gene	Lipid nanoparticle (proprietary lipid nanoparticle (LNP) delivery-system proprietary ionizable lipid, combined with a phospholipid, a pegylated lipid (molecular weight of polyethylene glycol, 2000 Da), and cholesterol)	Hereditary transthyretin amyloidosis	Phase 1, NCT04601051
CNM-Au8 (Nanocrystalline gold)	Gold nanocrystals	ALS	Phase 1, NCT04081714
CNM-Au8 (Nanocrystalline gold)	Gold nanocrystals	ALS	Phase 2, NCT04098406
CNM-Au8 (Nanocrystalline gold)	Gold nanocrystals	ALS	Phase 2, NCT03843710
CNM-Au8 (Nanocrystalline gold)	Gold nanocrystals	PD	Phase 2, NCT03815916

AD—Alzheimer’s disease; ALS—amyotrophic lateral sclerosis; DLin-MC3-DMA—dilinoleylmethyl-4-dimethylaminobutyrate; DSPC—distearoylphosphatidylcholine; PEG2000-C-DMG—1,2-dimyristoyl-rac-glycero-3-methoxypolyethylene glycol-2000; PD—Parkinson’s disease.

## Data Availability

Not applicable.

## References

[1] Merelli A., Czornyj L., Lazarowski A. (2013). Erythropoietin: A neuroprotective agent in cerebral hypoxia, neurodegeneration, and epilepsy. Curr. Pharm. Des..

[2] Choonara Y.E., Pillay V., Du Toit L.C., Modi G., Naidoo D., Ndesendo V.M., Sibambo S.R. (2009). Trends in the molecular pathogenesis and clinical therapeutics of common neurodegenerative disorders. Int. J. Mol. Sci..

[3] Rapp T., Chauvin P., Costa N., Molinier L. (2015). Health economic considerations in neurodegenerative disorders. Imaging Neurodegener. Disord..

[4] Harilal S., Jose J., Parambi D.G.T., Kumar R., Mathew G.E., Uddin M.S., Kim H., Mathew B. (2019). Advancements in nanotherapeutics for Alzheimer’s disease: Current perspectives. J. Pharm. Pharmacol..

[5] Hinge N.S., Kathuria H., Pandey M.M. (2022). Engineering of structural and functional properties of nanotherapeutics and nanodiagnostics for intranasal brain targeting in Alzheimer’s. Appl. Mater. Today.

[6] Montazersaheb S., Ahmadian E., Maleki Dizaj S., Jahanbani Y., Davaran S., Huseynova I., Zhdanov R., Keskin C., Khalilov R., Eftekhari A. (2021). Emerging Nanotherapeutic Strategies in Alzheimer’s Disease. Frontiers in Clinical Drug Research-Dementia.

[7] (2002). Brain Basics: The Life and Death of a Neuron.

[8] Van den Heuvel M.P., Sporns O. (2013). Network hubs in the human brain. Trends Cogn. Sci..

[9] Kempermann G. (2006). Adult Neurogenesis: Stem Cells and Neuronal Development in the Adult Brain.

[10] Pino A., Fumagalli G., Bifari F., Decimo I. (2017). New neurons in adult brain: Distribution, molecular mechanisms and therapies. Biochem. Pharmacol..

[11] Ganat Y.M., Silbereis J., Cave C., Ngu H., Anderson G.M., Ohkubo Y., Ment L.R., Vaccarino F.M. (2006). Early postnatal astroglial cells produce multilineage precursors and neural stem cells in vivo. J. Neurosci..

[12] Przedborski S., Vila M., Jackson-Lewis V. (2003). Series Introduction: Neurodegeneration: What is it and where are we?. J. Clin. Investig..

[13] Hoover B.R., Reed M.N., Su J., Penrod R.D., Kotilinek L.A., Grant M.K., Pitstick R., Carlson G.A., Lanier L.M., Yuan L.-L. (2010). Tau mislocalization to dendritic spines mediates synaptic dysfunction independently of neurodegeneration. Neuron.

[14] Milnerwood A.J., Raymond L.A. (2010). Early synaptic pathophysiology in neurodegeneration: Insights from Huntington’s disease. Trends Neurosci..

[15] Scott D.A., Tabarean I., Tang Y., Cartier A., Masliah E., Roy S. (2010). A pathologic cascade leading to synaptic dysfunction in α-synuclein-induced neurodegeneration. J. Neurosci..

[16] Kovacs G.G. (2019). Molecular pathology of neurodegenerative diseases: Principles and practice. J. Clin. Pathol..

[17] Martin J.B. (1999). Molecular basis of the neurodegenerative disorders. N. Engl. J. Med..

[18] Mattson M.P. (2000). Apoptosis in neurodegenerative disorders. Nat. Rev. Mol. Cell Biol..

[19] Hague S., Klaffke S., Bandmann O. (2005). Neurodegenerative disorders: Parkinson’s disease and Huntington’s disease. J. Neurol. Neurosurg. Psychiatry.

[20] Harding B.N., Kariya S., Monani U.R., Chung W.K., Benton M., Yum S.W., Tennekoon G., Finkel R.S. (2015). Spectrum of neuropathophysiology in spinal muscular atrophy type I. J. Neuropathol. Exp. Neurol..

[21] Klockgether T., Mariotti C., Paulson H.L. (2019). Spinocerebellar ataxia. Nat. Rev. Dis. Primers.

[22] Liu H., Hu Y., Zhang Y., Zhang H., Gao S., Wang L., Wang T., Han Z., Sun B.L., Liu G. (2022). Mendelian randomization highlights significant difference and genetic heterogeneity in clinically diagnosed Alzheimer’s disease GWAS and self-report proxy phenotype GWAX. Alzheimer’s Res. Ther..

[23] Jain N., Chen-Plotkin A.S. (2018). Genetic modifiers in neurodegeneration. Curr. Genet. Med. Rep..

[24] Jain V., Baitharu I., Barhwal K., Prasad D., Singh S.B., Ilavazhagan G.J.C. (2012). Enriched environment prevents hypobaric hypoxia induced neurodegeneration and is independent of antioxidant signaling. Cell. Mol. Neurobiol..

[25] Esch T., Stefano G.B., Fricchione G.L., Benson H.J.N.L. (2002). The role of stress in neurodegenerative diseases and mental disorders. Neuro Endocrinol. Lett..

[26] Allan S.M., Rothwell N.J. (2003). Inflammation in central nervous system injury. Philos. Trans. R. Soc. B Biol. Sci..

[27] Liu Z., Zhou T., Ziegler A.C., Dimitrion P., Zuo L. (2017). Oxidative stress in neurodegenerative diseases: From molecular mechanisms to clinical applications. Oxid. Med. Cell. Longev..

[28] Brouwer-DudokdeWit A.C., Savenije A., Zoeteweij M.W., Maat-Kievit A., Tibben A. (2002). A hereditary disorder in the family and the family life cycle: Huntington disease as a paradigm. Fam. Process.

[29] Bayer T.A., Wirths O. (2010). Intracellular accumulation of amyloid-Beta-a predictor for synaptic dysfunction and neuron loss in Alzheimer’s disease. Front. Aging Neurosci..

[30] Pickett E.K., Herrmann A.G., McQueen J., Abt K., Dando O., Tulloch J., Jain P., Dunnett S., Sohrabi S., Fjeldstad M.P. (2019). Amyloid beta and tau cooperate to cause reversible behavioral and transcriptional deficits in a model of Alzheimer’s disease. Cell Rep..

[31] Butterfield D.A., Griffin S., Munch G., Pasinetti G.M. (2002). Amyloid β-peptide and amyloid pathology are central to the oxidative stress and inflammatory cascades under which Alzheimer’s disease brain exists. J. Alzheimer’s Dis..

[32] Paula V.d.J.R.d., Guimarães F.M., Diniz B.S., Forlenza O.V. (2009). Neurobiological pathways to Alzheimer’s disease: Amyloid-beta, TAU protein or both?. Dement. Neuropsychol..

[33] Mondragón-Rodríguez S., Perry G., Zhu X., Boehm J. (2012). Amyloid Beta and tau proteins as therapeutic targets for Alzheimer’s disease treatment: Rethinking the current strategy. Int. J. Alzheimer’s Dis..

[34] Spires-Jones T.L., Hyman B.T. (2014). The intersection of amyloid beta and tau at synapses in Alzheimer’s disease. Neuron.

[35] Akhondzadeh S., Noroozian M. (2002). Alzheimer’s disease: Pathophysiology and pharmacotherapy. IDrugs Investig. Drugs J..

[36] Kouli A., Torsney K.M., Kuan W.-L. (2018). Parkinson’s disease: Etiology, neuropathology, and pathogenesis. Exon Publ..

[37] Spires-Jones T.L., Attems J., Thal D.R. (2017). Interactions of pathological proteins in neurodegenerative diseases. Acta Neuropathol..

[38] Priyadarshi A., Khuder S.A., Schaub E.A., Priyadarshi S.S. (2001). Environmental risk factors and Parkinson’s disease: A metaanalysis. Environ. Res..

[39] Emamzadeh F.N., Surguchov A. (2018). Parkinson’s disease: Biomarkers, treatment, and risk factors. Front. Neurosci..

[40] Gorell J.M., Peterson E.L., Rybicki B.A., Johnson C.C. (2004). Multiple risk factors for Parkinson’s disease. J. Neurol. Sci..

[41] Kuopio A.M., Marttila R.J., Helenius H., Rinne U.K. (1999). Environmental risk factors in Parkinson’s disease. Mov. Disord. Off. J. Mov. Disord. Soc..

[42] Bartels A.L., Leenders K.L. (2009). Parkinson’s disease: The syndrome, the pathogenesis and pathophysiology. Cortex.

[43] Chaudhuri K.R., Schapira A.H. (2009). Non-motor symptoms of Parkinson’s disease: Dopaminergic pathophysiology and treatment. Lancet Neurol..

[44] Moore D.J., West A.B., Dawson V.L., Dawson T.M. (2005). Molecular pathophysiology of Parkinson’s disease. Annu. Rev. Neurosci..

[45] Schulz-Schaeffer W.J. (2010). The synaptic pathology of α-synuclein aggregation in dementia with Lewy bodies, Parkinson’s disease and Parkinson’s disease dementia. Acta Neuropathol..

[46] Gendron T.F., Petrucelli L. (2009). The role of tau in neurodegeneration. Mol. Neurodegener..

[47] Morris J. (2015). Amyotrophic lateral sclerosis (ALS) and related motor neuron diseases: An overview. Neurodiagn. J..

[48] Wang G., Rayner S., Chung R., Shi B., Liang X. (2020). Advances in nanotechnology-based strategies for the treatments of amyotrophic lateral sclerosis. Mater. Today Bio..

[49] Hardiman O., Al-Chalabi A., Chio A., Corr E.M., Logroscino G., Robberecht W., Shaw P.J., Simmons Z., Van Den Berg L.H. (2017). Amyotrophic lateral sclerosis. Nat. Rev. Dis. Primers.

[50] Rossi F.H., Franco M.C., Estevez A.G. (2013). Pathophysiology of amyotrophic lateral sclerosis. Current Advances in Amyotrophic Lateral Sclerosis.

[51] Webster C.P., Smith E.F., Bauer C.S., Moller A., Hautbergue G.M., Ferraiuolo L., Myszczynska M.A., Higginbottom A., Walsh M.J., Whitworth A.J. (2016). The C9orf72 protein interacts with Rab1a and the ULK 1 complex to regulate initiation of autophagy. EMBO J..

[52] Dunn B., Stein P., Cavazzoni P. (2021). Approval of Aducanumab for Alzheimer Disease—The FDA’s Perspective. JAMA Intern. Med..

[53] Walsh S., Merrick R., Milne R., Brayne C. (2021). Aducanumab for Alzheimer’s disease?. BMJ.

[54] Wu K.-M., Zhang Y.-R., Huang Y.-Y., Dong Q., Tan L., Yu J.-T. (2021). The role of the immune system in Alzheimer’s disease. Ageing Res. Rev..

[55] Hampel H., Mesulam M.-M., Cuello A.C., Khachaturian A.S., Vergallo A., Farlow M., Snyder P., Giacobini E., Khachaturian Z. (2019). Revisiting the cholinergic hypothesis in Alzheimer’s disease: Emerging evidence from translational and clinical research. J. Prev. Alzheimer’s Dis..

[56] Deardorff W.J., Feen E., Grossberg G.T. (2015). The use of cholinesterase inhibitors across all stages of Alzheimer’s disease. Drugs Aging.

[57] Liu P.-P., Xie Y., Meng X.-Y., Kang J.-S. (2019). History and progress of hypotheses and clinical trials for Alzheimer’s disease. Signal Transduct. Target. Ther..

[58] Johnson J.W., Kotermanski S.E. (2006). Mechanism of action of memantine. Curr. Opin. Pharmacol..

[59] Reisberg B., Doody R., Stöffler A., Schmitt F., Ferris S., Möbius H. (2003). Memantine treatment in patients with moderate-to-severe AD. N. Engl. J. Med..

[60] Folch J., Busquets O., Ettcheto M., Sánchez-López E., Castro-Torres R.D., Verdaguer E., Garcia M.L., Olloquequi J., Casadesús G., Beas-Zarate C. (2018). Memantine for the treatment of dementia: A review on its current and future applications. J. Alzheimer’s Dis..

[61] Alexander G.C., Knopman D.S., Emerson S.S., Ovbiagele B., Kryscio R.J., Perlmutter J.S., Kesselheim A.S. (2021). Revisiting FDA Approval of Aducanumab. N. Engl. J. Med..

[62] Armstrong M.J., Okun M.S. (2020). Diagnosis and treatment of Parkinson disease: A review. JAMA.

[63] Bucchia M., Ramirez A., Parente V., Simone C., Nizzardo M., Magri F., Dametti S., Corti S. (2015). Therapeutic development in amyotrophic lateral sclerosis. Clin. Ther..

[64] Jaiswal M.K. (2019). Riluzole and edaravone: A tale of two amyotrophic lateral sclerosis drugs. Med. Res. Rev..

[65] Ballabh P., Braun A., Nedergaard M. (2004). The blood–brain barrier: An overview: Structure, regulation, and clinical implications. Neurobiol. Dis..

[66] Daneman R., Prat A. (2015). The blood–brain barrier. Cold Spring Harb. Perspect. Biol..

[67] Felgenhauer K. (1974). Protein size and cerebrospinal fluid composition. Klin. Wochenschr..

[68] Greene C., Campbell M. (2016). Tight junction modulation of the blood brain barrier: CNS delivery of small molecules. Tissue Barriers.

[69] Hawkins B.T., Davis T.P. (2005). The blood-brain barrier/neurovascular unit in health and disease. Pharmacol. Rev..

[70] Nakagawa S., Deli M.A., Kawaguchi H., Shimizudani T., Shimono T., Kittel A., Tanaka K., Niwa M. (2009). A new blood–brain barrier model using primary rat brain endothelial cells, pericytes and astrocytes. Neurochem. Int..

[71] Serlin Y., Shelef I., Knyazer B., Friedman A. (2015). Anatomy and physiology of the blood–brain barrier. Seminars in Cell & Developmental Biology.

[72] Begley D.J. (2004). ABC transporters and the blood-brain barrier. Curr. Pharm. Des..

[73] Hladky S.B., Barrand M.A. (2018). Elimination of substances from the brain parenchyma: Efflux via perivascular pathways and via the blood–brain barrier. Fluids Barriers CNS.

[74] Lockman P., Mumper R., Khan M., Allen D. (2002). Nanoparticle technology for drug delivery across the blood-brain barrier. Drug Dev. Ind. Pharm..

[75] Van de Waterbeemd H., Camenisch G., Folkers G., Chretien J.R., Raevsky O.A. (1998). Estimation of blood-brain barrier crossing of drugs using molecular size and shape, and H-bonding descriptors. J. Drug Target..

[76] Di L., Artursson P., Avdeef A., Ecker G.F., Faller B., Fischer H., Houston J.B., Kansy M., Kerns E.H., Krämer S.D. (2012). Evidence-based approach to assess passive diffusion and carrier-mediated drug transport. Drug Discov. Today.

[77] Fischer H., Gottschlich R., Seelig A. (1998). Blood-brain barrier permeation: Molecular parameters governing passive diffusion. J. Membr. Biol..

[78] Arora S., Sharma D., Singh J. (2020). GLUT-1: An effective target to deliver brain-derived neurotrophic factor gene across the blood brain barrier. ACS Chem. Neurosci..

[79] Lee H., Pienaar I.S. (2014). Disruption of the blood-brain barrier in Parkinson’s disease: Curse or route to a cure. Front. Biosci. Landmark Ed..

[80] Kook S.-Y., Seok Hong H., Moon M., Mook-Jung I. (2013). Disruption of blood-brain barrier in Alzheimer disease pathogenesis. Tissue Barriers.

[81] Argaw A.T., Asp L., Zhang J., Navrazhina K., Pham T., Mariani J.N., Mahase S., Dutta D.J., Seto J., Kramer E.G. (2012). Astrocyte-derived VEGF-A drives blood-brain barrier disruption in CNS inflammatory disease. J. Clin. Investig..

[82] Sengillo J.D., Winkler E.A., Walker C.T., Sullivan J.S., Johnson M., Zlokovic B.V. (2013). Deficiency in Mural Vascular Cells Coincides with Blood–Brain Barrier Disruption in A lzheimer’s Disease. Brain Pathol..

[83] Dong X. (2018). Current Strategies for Brain Drug Delivery. Theranostics.

[84] Ghuman J., Zunszain P.A., Petitpas I., Bhattacharya A.A., Otagiri M., Curry S. (2005). Structural basis of the drug-binding specificity of human serum albumin. J. Mol. Biol..

[85] Krol S. (2012). Challenges in drug delivery to the brain: Nature is against us. J. Control. Release Off. J. Control. Release Soc..

[86] Pardridge W.M. (2012). Drug transport across the blood-brain barrier. J. Cereb. Blood Flow Metab..

[87] Teleanu D.M., Negut I., Grumezescu V., Grumezescu A.M., Teleanu R.I. (2019). Nanomaterials for drug delivery to the central nervous system. Nanomaterials.

[88] Poovaiah N., Davoudi Z., Peng H., Schlichtmann B., Mallapragada S., Narasimhan B., Wang Q. (2018). Treatment of neurodegenerative disorders through the blood–brain barrier using nanocarriers. Nanoscale.

[89] Chauhan P.S., Yadav D., Koul B., Mohanta Y.K., Jin J.O. (2020). Recent Advances in Nanotechnology: A Novel Therapeutic System for the Treatment of Alzheimer’s Disease. Curr. Drug Metab..

[90] Spuch C., Saida O., Navarro C. (2012). Advances in the treatment of neurodegenerative disorders employing nanoparticles. Recent Pat. Drug Deliv. Formul..

[91] Modi G., Pillay V., Choonara Y.E. (2010). Advances in the treatment of neurodegenerative disorders employing nanotechnology. Ann. N. Y. Acad. Sci..

[92] Mignani S., Bryszewska M., Zablocka M., Klajnert-Maculewicz B., Cladera J., Shcharbin D., Majoral J.-P. (2017). Can dendrimer based nanoparticles fight neurodegenerative diseases? Current situation versus other established approaches. Prog. Polym. Sci..

[93] Dos Santos Rodrigues B., Lakkadwala S., Kanekiyo T., Singh J. (2020). Dual-modified liposome for targeted and enhanced gene delivery into mice brain. J. Pharmacol. Exp. Ther..

[94] Dos Santos Rodrigues B., Arora S., Kanekiyo T., Singh J. (2020). Efficient neuronal targeting and transfection using RVG and transferrin-conjugated liposomes. Brain Res..

[95] Sharma G., Modgil A., Layek B., Arora K., Sun C., Law B., Singh J. (2013). Cell penetrating peptide tethered bi-ligand liposomes for delivery to brain in vivo: Biodistribution and transfection. J. Control. Release.

[96] Cano A., Sánchez-López E., Ettcheto M., López-Machado A., Espina M., Souto E.B., Galindo R., Camins A., García M.L., Turowski P. (2020). Current advances in the development of novel polymeric nanoparticles for the treatment of neurodegenerative diseases. Nanomedicine.

[97] Fatima S., Quadri S.N., Parveen S., Beg S., Barkat M.A., Samim M., Abdin M., Ahmad F.J. (2021). Nanomedicinal Strategies as Emerging Therapeutic Avenues to Treat and Manage Cerebral Ischemia. CNS Neurol. Disord. Drug Targets.

[98] Vissers C., Ming G.L., Song H. (2019). Nanoparticle technology and stem cell therapy team up against neurodegenerative disorders. Adv. Drug Deliv. Rev..

[99] Salih N.A. (2013). The enhancement of breast cancer radiotherapy by using silver nanoparticles with 6 MeV gamma photons. Synthesis.

[100] Curry T., Kopelman R., Shilo M., Popovtzer R. (2014). Multifunctional theranostic gold nanoparticles for targeted CT imaging and photothermal therapy. Contrast Media Mol. Imaging.

[101] Khongkow M., Yata T., Boonrungsiman S., Ruktanonchai U.R., Graham D., Namdee K. (2019). Surface modification of gold nanoparticles with neuron-targeted exosome for enhanced blood–brain barrier penetration. Sci. Rep..

[102] Kim T., Lee N., Arifin D.R., Shats I., Janowski M., Walczak P., Hyeon T., Bulte J.W.M. (2016). In Vivo Micro-CT Imaging of Human Mesenchymal Stem Cells Labeled with Gold-Poly-L-Lysine Nanocomplexes. Adv. Funct. Mater..

[103] Jara-Guajardo P., Cabrera P., Celis F., Soler M., Berlanga I., Parra-Muñoz N., Acosta G., Albericio F., Guzman F., Campos M. (2020). Gold nanoparticles mediate improved detection of β-amyloid aggregates by fluorescence. Nanomaterials.

[104] Martins P.A.T., Alsaiari S., Julfakyan K., Nie Z., Khashab N.M. (2017). Self-assembled lipoprotein based gold nanoparticles for detection and photothermal disaggregation of β-amyloid aggregates. Chem. Commun..

[105] Masoudi Asil S., Ahlawat J., Guillama Barroso G., Narayan M. (2020). Nanomaterial based drug delivery systems for the treatment of neurodegenerative diseases. Biomater. Sci..

[106] Locatelli E., Naddaka M., Uboldi C., Loudos G., Fragogeorgi E., Molinari V., Pucci A., Tsotakos T., Psimadas D., Ponti J. (2014). Targeted delivery of silver nanoparticles and alisertib: In vitro and in vivo synergistic effect against glioblastoma. Nanomedicine.

[107] Wei M., Li S., Yang Z., Zheng W., Le W. (2017). Gold nanoparticles enhance the differentiation of embryonic stem cells into dopaminergic neurons via mTOR/p70S6K pathway. Nanomedicine.

[108] Aliev G., Daza J., Solís Herrera A., del Carmen Arias Esparza M., Morales L., Echeverria V., Bachurin S.O., Barreto G.E. (2015). Nanoparticles as Alternative Strategies for Drug Delivery to the Alzheimer Brain: Electron Microscopy Ultrastructural Analysis. CNS Neurol. Disord.-Drug Targets Former. Curr. Drug Targets-CNS Neurol. Disord..

[109] Huang C.L., Hsiao I.L., Lin H.C., Wang C.F., Huang Y.J., Chuang C.Y. (2015). Silver nanoparticles affect on gene expression of inflammatory and neurodegenerative responses in mouse brain neural cells. Environ. Res..

[110] Rajendran K., Anwar A., Khan N.A., Siddiqui R. (2017). Brain-eating amoebae: Silver nanoparticle conjugation enhanced efficacy of anti-amoebic drugs against Naegleria fowleri. ACS Chem. Neurosci..

[111] Gonzalez-Carter D.A., Leo B.F., Ruenraroengsak P., Chen S., Goode A.E., Theodorou I.G., Chung K.F., Carzaniga R., Shaffer M.S., Dexter D.T. (2017). Silver nanoparticles reduce brain inflammation and related neurotoxicity through induction of H(2)S-synthesizing enzymes. Sci. Rep..

[112] Skalska J., Strużyńska L. (2015). Toxic effects of silver nanoparticles in mammals—Does a risk of neurotoxicity exist?. Folia Neuropathol..

[113] Bony B.A., Kievit F.M. (2019). A Role for Nanoparticles in Treating Traumatic Brain Injury. Pharmaceutics.

[114] Tang J., Xiong L., Wang S., Wang J., Liu L., Li J., Yuan F., Xi T. (2009). Distribution, translocation and accumulation of silver nanoparticles in rats. J. Nanosci. Nanotechnol..

[115] Arya A., Gangwar A., Singh S.K., Roy M., Das M., Sethy N.K., Bhargava K. (2016). Cerium oxide nanoparticles promote neurogenesis and abrogate hypoxia-induced memory impairment through AMPK-PKC-CBP signaling cascade. Int. J. Nanomed..

[116] Pinzón-Daza M.L., Campia I., Kopecka J., Garzón R., Ghigo D., Riganti C. (2013). Nanoparticle- and liposome-carried drugs: New strategies for active targeting and drug delivery across blood-brain barrier. Curr. Drug Metab..

[117] Naz S., Beach J., Heckert B., Tummala T., Pashchenko O., Banerjee T., Santra S. (2017). Cerium oxide nanoparticles: A ‘radical’ approach to neurodegenerative disease treatment. Nanomedicine.

[118] Zavvari F., Nahavandi A., Shahbazi A. (2020). Neuroprotective effects of cerium oxide nanoparticles on experimental stress-induced depression in male rats. J. Chem. Neuroanat..

[119] Wu M.-R., Lee C.-H., Hsiao J.-K. (2020). Bidirectional enhancement of cell proliferation between iron oxide nanoparticle-labeled mesenchymal stem cells and choroid plexus in a cell-based therapy model of ischemic stroke. Int. J. Nanomed..

[120] Balducci C., Mancini S.A.-O., Minniti S., La Vitola P.A.-O., Zotti M., Sancini G., Mauri M., Cagnotto A., Colombo L.A.-O., Fiordaliso F. (2014). Multifunctional liposomes reduce brain β-amyloid burden and ameliorate memory impairment in Alzheimer’s disease mouse models. J. Neurosci..

[121] Arora S., Layek B., Singh J. (2020). Design and validation of liposomal ApoE2 gene delivery system to evade blood–brain barrier for effective treatment of Alzheimer’s disease. Mol. Pharm..

[122] Arora S., Singh J. (2021). In vitro and in vivo optimization of liposomal nanoparticles based brain targeted vgf gene therapy. Int. J. Pharm..

[123] Zhou H., S Beevers C., Huang S. (2011). The targets of curcumin. Curr. Drug Targets.

[124] Yanagisawa D., Amatsubo T., Morikawa S., Taguchi H., Urushitani M., Shirai N., Hirao K., Shiino A., Inubushi T., Tooyama I. (2011). In vivo detection of amyloid β deposition using ¹⁹F magnetic resonance imaging with a ¹⁹F-containing curcumin derivative in a mouse model of Alzheimer’s disease. Neuroscience.

[125] Lee I., Yang J., Lee J.H., Choe Y.S. (2011). Synthesis and evaluation of 1-(4-[¹⁸F]fluoroethyl)-7-(4’-methyl)curcumin with improved brain permeability for β-amyloid plaque imaging. Bioorganic Med. Chem. Lett..

[126] Bhatt R., Singh D., Prakash A., Mishra N. (2015). Development, characterization and nasal delivery of rosmarinic acid-loaded solid lipid nanoparticles for the effective management of Huntington’s disease. Drug Deliv..

[127] Sharma D., Singh J. (2020). Long-term glycemic control and prevention of diabetes complications in vivo using oleic acid-grafted-chitosan-zinc-insulin complexes incorporated in thermosensitive copolymer. J. Control. Release.

[128] Baba M., Itaka K., Kondo K., Yamasoba T., Kataoka K. (2015). Treatment of neurological disorders by introducing mRNA in vivo using polyplex nanomicelles. J. Control. Release.

[129] Arora S., Trivedi R., Lamptey R.N., Chaulagain B., Layek B., Singh J. (2021). Smart biopolymers for controlled drug delivery applications. Tailor-Made and Functionalized Biopolymer Systems.

[130] Kim J.-Y., Choi W.I., Kim Y.H., Tae G. (2013). Brain-targeted delivery of protein using chitosan-and RVG peptide-conjugated, pluronic-based nano-carrier. Biomaterials.

[131] Layek B., Singh J. (2013). Amino acid grafted chitosan for high performance gene delivery: Comparison of amino acid hydrophobicity on vector and polyplex characteristics. Biomacromolecules.

[132] Xue Y., Wang N., Zeng Z., Huang J., Xiang Z., Guan Y.-Q. (2020). Neuroprotective effect of chitosan nanoparticle gene delivery system grafted with acteoside (ACT) in Parkinson’s disease models. J. Mater. Sci. Technol..

[133] Jaruszewski K.M., Ramakrishnan S., Poduslo J.F., Kandimalla K.K. (2012). Chitosan enhances the stability and targeting of immuno-nanovehicles to cerebro-vascular deposits of Alzheimer’s disease amyloid protein. Nanomed. Nanotechnol. Biol. Med..

[134] Cai Q., Wang L., Deng G., Liu J., Chen Q., Chen Z. (2016). Systemic delivery to central nervous system by engineered PLGA nanoparticles. Am. J. Transl. Res..

[135] Bhatt P.C., Verma A., Al-Abbasi F.A., Anwar F., Kumar V., Panda B.P. (2017). Development of surface-engineered PLGA nanoparticulate-delivery system of Tet1-conjugated nattokinase enzyme for inhibition of Aβ(40) plaques in Alzheimer’s disease. Int. J. Nanomed..

[136] Carradori D., Balducci C., Re F., Brambilla D., Le Droumaguet B., Flores O., Gaudin A., Mura S., Forloni G., Ordoñez-Gutierrez L. (2018). Antibody-functionalized polymer nanoparticle leading to memory recovery in Alzheimer’s disease-like transgenic mouse model. Nanotechnol. Biol. Med..

[137] Saffari P.M., Alijanpour S., Takzaree N., Sahebgharani M., Etemad-Moghadam S., Noorbakhsh F., Partoazar A. (2020). Metformin loaded phosphatidylserine nanoliposomes improve memory deficit and reduce neuroinflammation in streptozotocin-induced Alzheimer’s disease model. Life Sci..

[138] Amini S., Saudi A., Amirpour N., Jahromi M., Najafabadi S.S., Kazemi M., Rafienia M., Salehi H. (2020). Application of electrospun polycaprolactone fibers embedding lignin nanoparticle for peripheral nerve regeneration: In vitro and in vivo study. Int. J. Biol. Macromol..

[139] Mili B., Das K., Kumar A., Saxena A., Singh P., Ghosh S., Bag S. (2018). Preparation of NGF encapsulated chitosan nanoparticles and its evaluation on neuronal differentiation potentiality of canine mesenchymal stem cells. J. Mater. Sci. Mater. Med..

[140] Zielińska A., Costa B., Ferreira M.V., Miguéis D., Louros J., Durazzo A., Lucarini M., Eder P., V Chaud M., Morsink M. (2020). Nanotoxicology and nanosafety: Safety-by-design and testing at a glance. Int. J. Environ. Res. Public Health.

[141] Hofmann-Amtenbrink M., Hofmann H., Hool A., Roubert F. (2014). Nanotechnology in medicine: European research and its implications. Swiss Med. Wkly..

[142] Suk J.S., Xu Q., Kim N., Hanes J., Ensign L.M. (2016). PEGylation as a strategy for improving nanoparticle-based drug and gene delivery. Adv. Drug Deliv. Rev..

[143] Neves A., van der Putten L., Queiroz J., Pinheiro M., Reis S. (2021). Transferrin-functionalized lipid nanoparticles for curcumin brain delivery. J. Biotechnol..

[144] Ji X., Lu W., Wu K., Cho W.C. (2017). Influencing Factors of the Pharmacokinetic Characters on Nanopharmaceutics. Pharm. Nanotechnol..

[145] Souto E.B., Silva G.F., Dias-Ferreira J., Zielinska A., Ventura F., Durazzo A., Lucarini M., Novellino E., Santini A. (2020). Nanopharmaceutics: Part II—Production scales and clinically compliant production methods. Nanomaterials.

[146] Zhu F.-D., Hu Y.-J., Yu L., Zhou X.-G., Wu J.-M., Tang Y., Qin D.-L., Fan Q.-Z., Wu A.-G. (2021). Nanoparticles: A Hope for the Treatment of Inflammation in CNS. Front. Pharmacol..

